# P329: Traditional medicine therapy a paradigm shift in promotion of gerontological sexual medicine in sub-Saharan African countries

**DOI:** 10.1186/2047-2994-2-S1-P329

**Published:** 2013-06-20

**Authors:** K Odor, R Opara, N Iwuji

**Affiliations:** 1 Health Promotion and Education, university of Ibadan, Nigeria, Ibadan, Nigeria; 2 Aged Care, Gertrude abbot Nursing Home, Sydney, Australia; 3 Immunization, Olive Hospital and Maternity, Aba, Nigeria

## Introduction

Traditional Medicine (TM) has responded to healthcare-delivery needs of most Africans over the years. However, TM means different things to different people. Single medicinal plant may be classified as food or herbal medicine, depending on the contest. However, lack of regulation means there are many fake remedies and false practitioners as genuine treatments.

## Objectives

This study was to examine Traditional Medicine (TM) therapy as a sexual medicine in sub-Saharan Africa.

## Methods

The study adopted quantitative and qualitative methods of data-collection. It was descriptively cross-sectional in design, comprising 800-respondents selected by multi-stage sampling technique. Twelve Focus Group Discussions (FGDs) and questionnaires data were analysed thematically and statistically respectively.

## Results

A total of 20.5% of participants under herbal-concoction engaged in extra-marital sex, while (5.8%) used TM to prevent infection during sex. Few (3.0%) used herbs/concoction (6.3%) to increase sexual performance. Moreover, (1.5%) suggested that herbal concoction could improve sexual health. Most (60.3%) postulated that visiting traditional healers; herbal-use (10.3%) and taking drugs (17.3%) would provide prevention/treatment against STDs. Majority of FGD participants believed in TM efficacy than orthodox-medicine. Hence, Magun could detect/prevent diseases. FGD participants opined that TM for-instance: Ale or Erii-in; Ajidewe and Agunmu are for erection; Ale is for ejaculation and sperm-production, Aseje and Afaatoo enhance multiple sexual-rounds, Agbo or Ogbolo and Tude boost sexual-performance.

## Conclusion

Most elderly in Nigeria used TM to enhance sexual performance. However, these products are yet to undergo clinical evaluation for sexual medicine. There is need to support its clinical investigation especially the claims in the improvement of sexual health.

## Disclosure of interest

None declared.

